# Access to a polymerase chain reaction assay method targeting 13 respiratory viruses can reduce antibiotics: a randomised, controlled trial

**DOI:** 10.1186/1741-7015-9-44

**Published:** 2011-04-26

**Authors:** Robin Brittain-Long, Johan Westin, Sigvard Olofsson, Magnus Lindh, Lars-Magnus Andersson

**Affiliations:** 1 Department of Infectious Diseases, Sahlgrenska University Hospital, Smörslottsgatan 1, SE-416 85, Gothenburg, Sweden; 2 Department of Clinical Virology, Sahlgrenska University Hospital, Guldhedsgatan 10 B, SE-413 46, Gothenburg, Sweden

## Abstract

**Background:**

Viral respiratory infections are common worldwide and range from completely benign disease to life-threatening illness. Symptoms can be unspecific, and an etiologic diagnosis is rarely established because of a lack of suitable diagnostic tools. Improper use of antibiotics is common in this setting, which is detrimental in light of the development of bacterial resistance. It has been suggested that the use of diagnostic tests could reduce antibiotic prescription rates. The objective of this study was to evaluate whether access to a multiplex polymerase chain reaction (PCR) assay panel for etiologic diagnosis of acute respiratory tract infections (ARTIs) would have an impact on antibiotic prescription rate in primary care clinical settings.

**Methods:**

Adult patients with symptoms of ARTI were prospectively included. Nasopharyngeal and throat swabs were analysed by using a multiplex real-time PCR method targeting thirteen viruses and two bacteria. Patients were recruited at 12 outpatient units from October 2006 through April 2009, and samples were collected on the day of inclusion (initial visit) and after 10 days (follow-up visit). Patients were randomised in an open-label treatment protocol to receive a rapid or delayed result (on the following day or after eight to twelve days). The primary outcome measure was the antibiotic prescription rate at the initial visit, and the secondary outcome was the total antibiotic prescription rate during the study period.

**Results:**

A total sample of 447 patients was randomised. Forty-one were excluded, leaving 406 patients for analysis. In the group of patients randomised for a rapid result, 4.5% (9 of 202) of patients received antibiotics at the initial visit, compared to 12.3% (25 of 204) (*P *= 0.005) of patients in the delayed result group. At follow-up, there was no significant difference between the groups: 13.9% (28 of 202) in the rapid result group and 17.2% (35 of 204) in the delayed result group (*P *= 0.359), respectively.

**Conclusions:**

Access to a rapid method for etiologic diagnosis of ARTIs may reduce antibiotic prescription rates at the initial visit in an outpatient setting. To sustain this effect, however, it seems necessary to better define how to follow and manage the patient according to the result of the test, which warrants further investigation.

**Trial registration:**

ClinicalTrials.gov identifier: NCT01133782.

## Background

Acute respiratory tract infections (ARTIs) represent a major global health burden [1], and viruses cause a large proportion of ARTIs. Distinguishing bacterial ARTIs that require antibiotic treatment from viral ARTIs not needing an antibiotic prescription can be difficult on clinical grounds alone and causes unnecessary use of antibiotics, with the highest rates occurring in the primary care setting [2,3]. Excess use of antibiotics has major implications for health economics and, more importantly, for the development of bacterial resistance [3,4], as well as for the individual patient in terms of adverse events such as allergic reactions and antibiotic-associated diarrhoea [5,6]. The predictive value of vital signs, C-reactive protein (CRP) and X-ray findings for diagnosing pneumonia requiring antibiotics is low [7,8]. The treatment of acute bronchitis serves as an illustrative example of the unnecessary use of antibiotics, where the recommended therapy for immune competent adults does not include antibiotic treatment [9,10], yet antibiotics were found to have been prescribed for this condition at high rates in studies conducted in the United Kingdom (64%) [11], Sweden (50% to 60%) [12,13] and the United States (59%) [14].

The use of improved diagnostic methods such as nucleic acid amplification tests (NAATs), including multiplex real-time polymerase chain reaction (RT-PCR) assays, has increased in recent years. These methods have proven to be equivalent or superior to conventional methods [15-19] and to have a short turnaround time at the laboratory, as well as affording clinicians the ability to analyse several respiratory agents within the same patient sample.

It has been suggested that the use of NAATs, including multiplex PCR methods, for the detection of respiratory pathogens could reduce antibiotic prescription rates [14,18]. The present study was designed to evaluate whether access to a multiplex RT-PCR method targeting thirteen viruses would have an impact on antibiotic prescription rates for ARTI in a primary care setting.

## Methods

### Study design

We conducted an investigator-initiated, multicentre, prospective, randomised, controlled trial with adult patients in a primary care setting. Eligibility criteria for participants were age ≥ 18 years and a diagnosis of community-acquired ARTI, defined as having a history of at least two of the following symptoms: coryza/nasal congestion/sneezing, sore throat/odynophagia, cough, pleuritic chest pain, shortness of breath or fever for which the physician found no other explanation, with a duration of less than 14 days. Exclusion criteria included confirmed bacterial infection (defined as a positive *Streptococcus *group A quick test and clinical findings corresponding to bacterial tonsillitis, perforated acute otitis media, high suspicion of lobar pneumococcal pneumonia or severe septicaemia, a positive blood culture for a clinically significant bacterial pathogen and clinical findings corresponding to septicaemia) and ongoing antibiotic treatment. Patients were recruited at 12 outpatient units (eight primary healthcare centres and four departments of infectious diseases), and samples were collected from October 2006 through April 2009. Signs and symptoms were recorded in a web-based case report form.

### Randomisation and masking

Patients were enrolled by the treating physician on the day of inclusion and stratified according to duration of symptoms of either ≤5 days or > 5 days. Open-label (nonblinded) randomisation (ratio 1:1) was performed by means of a predefined list and using a concealed, central, web-based procedure on the day of inclusion for the treating physician to receive the results from the multiplex PCR analysis either on the day following inclusion (the rapid result cohort) or eight to twelve days later (the delayed result cohort).

Recruitment was performed from Sunday through Thursday from 8 AM until 5 PM, allowing for the laboratory to report results the following day. Nasopharyngeal (flocked nylon swabs; Microrheologics, Brescia, Italy) and throat swab specimens were collected from each patient. The swabs were jointly placed in a sterile container with 1 mL of sodium chloride solution and sent to the laboratory the same day. Specimens were either analyzed directly or frozen at -70°C for delayed analysis (see randomisation and masking above). The results were communicated through the web-based case report form to a study nurse at each site. Additional diagnostic testing, including throat and sputum cultures, CRP and X-ray investigations, were left to the discretion of the treating physician and recorded in the case report form.

### Outcome measures

The objective of the study was to evaluate whether access to a rapid etiologic diagnostic method would have an impact on antibiotic prescription. The primary outcome measure was the antibiotic prescription rate at the initial visit (or within 48 hours thereafter), and the secondary outcome measure was the total antibiotic prescription rate during the study period. Antibiotic prescription at the initial visit (or within 48 hours thereafter) was recorded and analysed in relation to access to a rapid vs. a delayed result. Results in the rapid result group were provided to the treating physician within 24 hours for the majority of patients and within 48 hours for all patients. The final management of all patients and how to act upon the given result of the PCR assay were left to the discretion of the treating physician. All patients were asked to return for a follow-up visit eight to twelve days after the initial visit, and this time period represents the duration of follow-up in the study. The total antibiotic prescription rate (prescriptions at initial visit, at follow-up visit or between those visits) was recorded and constituted the secondary outcome measure in the study. The prescription of antiviral medications was not recorded. Serious adverse events (SAEs) were defined as death, life-threatening events, hospitalisation or events resulting or threatening to result in persistent or significant disability. The Regional Ethical Review board approved the study, and all patients provided written informed consent to participate in the study.

### Nucleic acid extraction and RT-PCR

We utilized a RT-PCR procedure based on automated specimen extraction and multiplex amplification adapted for respiratory specimens as previously described [20]. Briefly, nucleic acid from 100 μL of the respiratory specimen was extracted into an elution volume of 100 μL by using a Magnapure LC robot (Roche Molecular Systems, Mannheim, Germany) according to the total nucleic acid protocol and was amplified in an ABI 7500 Real-Time PCR System (Applied Biosystems, Foster City, CA, USA) in 50-μL reaction volumes. After a reverse transcription step, 45 cycles of two-step PCR were performed. Each sample was amplified in six parallel reactions containing primers and probes as previously reported [20], with the modification of adenovirus being analysed in a separate reaction. Included in the panel were parainfluenzavirus (PIV) types 1 through 3, influenza virus A (IfA) and influenza virus B (IfB), human metapneumovirus, respiratory syncytial virus (RSV), human rhinovirus (hRV), enterovirus (EV), adenovirus (AdV) and human coronavirus (hCoV) types 229E, OC43 and NL63, along with the bacteria *Mycoplasma pneumoniae *and *Chlamydophila pneumoniae*.

The primers and probes of all RT-PCR assays were designed to bind conserved segments of the targeted agents. This is particularly important for AdV, hRV and EV, which are characterised by a large number of subtypes. The accuracy of the AdV PCR assay has been documented by Heim *et al*. [21]. The target region for the hRV and EV assays was the conserved segment of the 5' untranslated region that allows amplification of all subtypes and which has been used previously by others [22-24]. The primers and probes used for IfB and PIV type 3 were developed by Dr Lars Nielsen, Copenhagen, Denmark; those for PIV types 1 and 2 were previously described by Watzinger *et al*. [22]; those for hCoV (types NL63, 229E and OC43) were described previously by Gunson *et al*. [24]; and those for IfA virus were a modification of a system published by Ward *et al*. [25].

### Statistical analysis

On the basis of the nature of the primary outcome measure, patients with protocol violations and/or missing data were excluded from the primary analysis. This was predefined in the analysis plan. The χ^2 ^test was used to compare proportions. *P *< 0.05 was considered statistically significant. The study was scheduled to include at least 200 patients in each group, allowing for a statistical power of 80% to demonstrate an estimated reduction in antibiotic prescription rate from 20% to 10% in the rapid result group. Multivariate analysis using backward stepwise (Wald test) logistic regression was carried out to analyse factors separately and independently to predict a positive PCR result as well as the prescription of antibiotics. SPSS version 17.0 for Macintosh software (SPSS Inc, Chicago, IL, USA) was used for all statistical analyses.

## Results

### Study design and baseline characteristics

**Figure 1:**
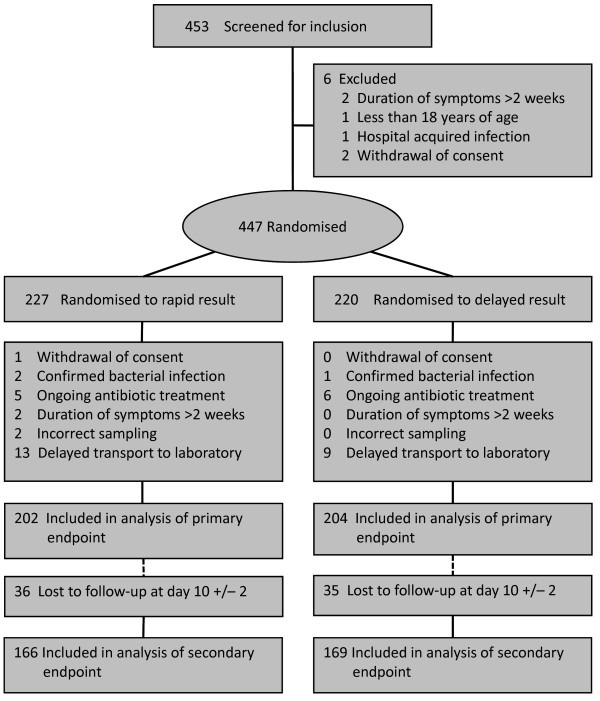
**Flowchart of patients**.

The patient flow according to the study design is shown in Figure 1. The baseline characteristics of the two groups of patients randomised for rapid vs. delayed results are shown in Table 1.

**Table 1:** Baseline characteristics of patients with acute respiratory tract infection openly randomised to rapid (within 24 to 48 hours) or delayed (after eight to twelve days) multiplex PCR assay results^a^

Variable	Rapid analysis group (*n *= 202)	Delayed analysis group (*n *= 204)
Demographics		
Median age, yr (IQR)	39 (31 to 50)	39 (31 to 53)
Male sex, *n *(%)	79 (39.1)	90 (44.1)
Coexisting illnesses^b^, *n *(%)		
Patients with available data	120 (59.4)	109 (53.4)
No reported coexisting illness	77 (64.2^c^)	68 (62.4^d^)
Asthma	14 (11.7^c^)	11 (10.1^d^)
COPD	2 (1.7^c^)	-
Allergies	6 (5.0^c^)	10 (9.2^d^)
Diabetes	2 (1.7^c^)	1 (0.9^d^)
Neoplastic disease	1 (0.8^c^)	2 (1.8^d^)
Autoimmune disease^e^	3 (2.5^c^)	4 (3.7^d^)
Ischaemic heart disease/angina	2 (1.7^c^)	1 (0.9^d^)
Clinical findings		
Median duration of symptoms, days (IQR)	5 (3 to 7)	5 (3 to 9)
Median body temperature, °C (IQR)	37.1 (36.6 to 37.5)	36.9 (36.6 to 37.4)
Body temperature ≥38.5°C, *n *(%)	6 (3.0^f^)	12 (6.0^g^)
Median heart rate, beats/minute (IQR)	76 (67 to 88)	78 (70 to 87)
Tachycardia^h^, *n *(%)	19 (9.4)	18 (8.8)
Median respiratory rate, breaths/min (IQR)	16 (14 to 19)	16 (14 to 19)
Hypoxia^i^, *n *(%)	9 (4.5)	8 (3.9)
Laboratory findings		
CRP < 50 mg/L, *n *(%)	122 (83.6^j^)	140 (90.3^k^)
CRP ≥50 mg/L, *n *(%)	24 (16.4^j^)	15 (9.7^k^)
Symptoms, *n *(%)		
Coryza	167 (82.7)	171 (83.8)
Sore throat	153 (75.7)	157 (77.0)
Headache	149 (74.1)	149 (73.0)
Dry cough	130 (67.0)	132 (67.0)
Productive cough	120 (59.4)	108 (52.9)
Shortness of breath	111 (55.0)	113 (55.4)
Fever	106 (52.7)	109 (53.4)
Myalgia	107 (53.0)	100 (49.0)
Red eyes	83 (41.1)	81 (39.9)
Joint pain	83 (41.1)	79 (38.7)
Chest pain	53 (26.2)	40 (19.6)
Diarrhoea	17 (8.4)	21 (10.3)
Vomiting	10 (5.0)	16 (7.8)
Rash	10 (5.0)	13 (6.4)

^a^IQR, interquartile range; COPD, chronic obstructive pulmonary disease; CRP, C-reactive protein; ^b^coexisting illnesses are those likely to influence the decision whether to prescribe antibiotics; conditions not included were fibromyalgia, lumbago, adenoids, migraine, depression, Parkinson's disease, hypothyroidism, osteoarthritis, Gilbert's syndrome, psoriasis, bradycardia, hepatitis C virus, scoliosis and pregnancy; ^c^number of patients with data for coexisting illness, rapid analysis group (*n *= 120); ^d^number of patients with data for coexisting illness, delayed analysis group (*n *= 109); ^e^autoimmune diseases included inflammatory bowel syndrome (Crohn's disease, ulcerative colitis), systemic lupus erythematosus, rheumatoid arthritis, polymyalgia rheumatica and multiple sclerosis; ^f^number of patients tested for body temperature, rapid analysis group (*n *= 198); ^g^number of patients tested for body temperature, delayed analysis group (*n *= 201); ^h^tachycardia defined as heart rate ≥100 beats/minute; ^i^hypoxia defined as pulse oximetry < 95%; ^j^number of patients tested for CRP, rapid analysis group (*n *= 146); ^k^number of patients tested for CRP, delayed analysis group (*n *= 155).

### Primary outcome measure

In the entire study population, antibiotics were prescribed for 8.4% (34 of 406) of patients at the initial visit (or within 48 hours thereof). In the group of patients randomised for a rapid result, 4.5% (9 of 202) of patients received an antibiotic, compared to 12.3% (25 of 204) of patients in the delayed PCR-based result group (*P *= 0.005) (see Table 2). Patients with symptom duration ≤ 5 days in the rapid result group received significantly fewer antibiotic prescriptions than patients in the delayed result group.

**Table 2:** Antibiotic prescription at initial visit (and within 48 hours of initial visit) for adult patients with acute respiratory tract infection, according to randomisation group (rapid result vs. delayed result)

Antibiotic prescription	Rapid result (*n *= 202)	Delayed result (*n *= 204)	*P*
Initial antibiotic treatment, *n *(%)	9 (4.5)	25 (12.3)	0.005^a^
At initial visit	7 (3.5)	21 (10.3)	
After 24 to 48 hours	2 (1.0)	4 (2.0)	
β-lactam^b^	4 (2.0)	13 (6.4)	-
Tetracycline	4 (2.0)	8 (3.9)	-
Macrolide	1 (0.5)	3 (1.5)	-
Quinolone	-	1 (0.5)	-
Patient demographics at initial antibiotic treatment, *n *(%)			
Body temperature ≥38.5°C, *n *(%)	-	4 (33.3^c^)	-
CRP level ≥50 mg/L, *n *(%)	2 (8.3^d^)	10 (67.0^e^)	< 0.001^a^
Duration of illness ≤5 days, *n *(%)	3 (3.3^f^)	12 (12.4^f^)	0.02^a^
Duration of illness > 5 days, *n *(%)	6 (5.4)	13 (12.1)	-
Patients with virus detected, *n *(%)	91 (45.0)	91 (44.6)	-
Antibiotics prescribed	3 (3.3^g^)	11 (12.1^g^)	0.03^a^
Patients with *Mycoplasma pneumoniae *detected, *n *(%)	5 (2.5)	2 (1.0)	-
Antibiotics prescribed, *n*	2^h^	2^h^	
Patients with *Chlamydophila pneumoniae *detected, *n *(%)	1 (0.5)	-	
Antibiotics prescribed, *n*	1^i^	-	-

^a^χ^2 ^test; ^b^phenoxymethylpenicillin or amoxicillin with or without clavulanic acid or loracarbef; ^c^four (33.3%) of twelve patients; ^d^two (8.3%) of twenty-four patients; ^e^10 (67.0%) of 15 patients; ^f^three (3.3%) of ninety-one patients and 12 (12.4%) of 97 patients, respectively; ^g^three (3.3%) of ninety-one patients and 11 (12.1%) of 91 patients, respectively; ^h^one patient received antibiotics within 24 hours and the other within 48 hours in each group, for a total of two patients in each group as indicated in table; ^i^patient received antibiotics within 48 hours.

Of the 34 patients who received initial antibiotic treatment, 14 (41%) tested positive for a respiratory virus, comprising three in the rapid result group and eleven in the delayed result group (see Table 2).

### Secondary outcome measure

A total of 335 (83%) of 406 patients returned for the optional follow-up visit or were available for a telephone appointment (visit, *n *= 243; telephone appointment, *n *= 92), comprising 166 (82%) of 202 patients in the rapid result group and 169 (83%) of 204 patients in the delayed result group. In total, 28 patients (13.9%) in the rapid result group and 35 patients (17.2%) in the delayed result group received antibiotic treatment at either the initial or follow-up visit. This difference was not statistically significant (*P *= 0.359). Antibiotic prescriptions outside the study (that is, by other than the study physician) were allowed. At the follow-up visit, two (11%) of nineteen patients in the rapid result group and one (11%) of nine patients in the delayed result group reported ongoing antibiotic treatment prescribed outside the study. The investigators reported no SAEs.

### Aetiology

As shown in Table 3, 191 patients (47%) tested positive for one agent on the basis of a multiplex PCR assay performed at the initial visit. In addition, 12 patients (5.9%) tested positive for two agents in the same sample. In three of these patients, one virus and one bacterium were detected, and in nine patients, two viruses were detected (see Table 4).

**Table 3:** Results (multiple detections not included) of multiplex real-time polymerase chain reaction assays of all included patients in order of frequency and by randomisation group (rapid vs. delayed result group)

Detected pathogens	All patients, *n *(%)	Rapid result group, *n *(%)	Delayed result group, *n *(%)
Influenza A virus	56 (13.8)	31 (15.3)	25 (12.3)
Rhinovirus	40 (9.9)	24 (11.9)	16 (7.8)
Coronavirus (all subtypes)	29 (7.1)	11 (5.4)	18 (8.8)
Coronavirus OC43	16 (3.9)	4 (2.0)	12 (5.9)
Coronavirus NL63	11 (2.7)	5 (2.5)	6 (2.9)
Coronavirus 229E	2 (0.5)	2 (1.0)	-
Respiratory syncytial virus	18 (4.4)	6 (3.0)	12 (5.9)
Influenza B virus	14 (3.4)	7 (3.5)	7 (3.4)
Metapneumovirus	14 (3.4)	6 (3.0)	8 (3.9)
Parainfluenzavirus types 1 through 3	7 (1.7)	4 (2.0)	3 (1.5)
*Mycoplasma pneumoniae*	7 (1.7)	5 (2.5)	2 (1.0)
Adenovirus	4 (1.0)	2 (1.0)	2 (1.0)
Enterovirus	1 (0.2)	-	1 (0.5)
*Chlamydophila pneumoniae*	1 (0.2)	1 (0.5)	-
No pathogen found	215 (53.0)	105 (52.0)	110 (54)
Total, *N *(%)	406 (100)	202 (100)	204 (100)

**Table 4:** Codetection of agents in multiplex real-time polymerase chain reaction assays from the same nasopharyngeal/oropharyngeal sample at initial visit of adults with ARTI^a ^by randomisation group (rapid result vs. delayed result)

Agent 1	Agent 2	*C*_t _value^b ^(agent 1/agent 2)
Rapid result group		
Rhinovirus	Influenza A virus	24/41
Rhinovirus	Respiratory syncytial virus	33/34
Rhinovirus	*Mycoplasma pneumoniae*	24/30
*Mycoplasma pneumoniae*	Rhinovirus	34/36
*Mycoplasma pneumoniae*	Coronavirus OC43	28/35
Adenovirus	Rhinovirus	20/35
Influenza B virus	Rhinovirus	36/38
Metapneumovirus	Rhinovirus	37/42
Parainfluenzavirus	Respiratory syncytial virus	28/30
Delayed result group		
Influenza A virus	Coronavirus OC43	29/31
Influenza B virus	Rhinovirus	31/37
Respiratory syncytial virus	Rhinovirus	31/36

^a^ARTI, acute respiratory tract infection; ^b^*C*_t _value, cycle threshold value.

### CRP levels

CRP levels were recorded in 301 (74%) of 406 patients, and a predefined subgroup analysis revealed that 39 patients (13%) had a CRP level ≥50 mg/L and that 15 patients (5%) had a CRP level > 100 mg/L. Of the 39 patients with a CRP level ≥50 mg/L, 12 patients (31%) received antibiotic treatment at the initial visit, compared to 17 (7%) of 262 patients in the group with a CRP level < 50 mg/L (*P *< 0.0001). In the rapid result group, two (8%) of twenty-four patients with a CRP level ≥50 mg/L received antibiotics, compared to 10 (67%) of 15 patients in the delayed result group (*P *= 0.0001). Patients who tested positive for *Mycoplasma pneumoniae *or *Chlamydophila pneumoniae *were included in the subgroup analysis of CRP levels (values for *M. pneumoniae*, available for five of seven patients, ranged from 58 mg/L to 210 mg/L, and the CRP level was 35 mg/L for the only patient who tested positive for *C. pneumoniae*). A virus (*M. pneumoniae *and *C. pneumoniae *excluded) was found in 49% of patients (19 of 39) with a CRP level ≥50 mg/L and in 27% of patients (4 of 15) with a CRP level ≥100 mg/L.

### Symptoms

In multivariate analysis of recorded symptoms, fever (odds ratio (OR) 1.98, 95% confidence interval (95% CI) 1.30 to 3.03, *P *= 0.002) and pleuritic chest pain (OR 1.72, 95% CI 1.04 to 2.86, *P *= 0.04) remained independently predictive of a positive PCR result (with *M. pneumoniae *and *C. pneumoniae *excluded from the analysis). A reported sore throat (OR 0.60, 95% CI 0.37 to 0.97, *P *= 0.04) was significantly more common among patients with a negative PCR result.

Vomiting remained the only symptom associated with the prescription of antibiotics at the initial visit (OR 5.91, 95% CI 2.20 to 15.85, *P *= 0.0004) in multivariate analysis. High fever (≥38.5°C) was more common in the group of patients randomised for a delayed result (12 of 201 patients; 6.0%) than in the group randomised for a rapid result (6 of 198; 3.0%) (see Table 1), but no significant difference in the number of antibiotic prescriptions at the initial visit for these groups was noted (see Table 2).

## Discussion

We have shown that access to a rapid result using a method for aetiologic diagnosis of ARTIs in an outpatient setting significantly reduced antibiotic prescriptions at the initial visit. However, this effect was no longer significant at the time of follow-up. In our study, we evaluated the impact of access to a rapid diagnostic tool rather than the impact of the actual test result, which implies that the mere prospect of a rapid aetiologic diagnosis can influence therapeutic decisions made for patients with an indistinct clinical presentation.

In the subgroup of patients with a CRP level ≥50 mg/L, this effect was even more pronounced. Among patients with positive PCR results for viruses, significantly fewer patients in the rapid result group received antibiotics than in the delayed result group. Our results are in line with the reduction of antibiotic prescriptions when rapid diagnostic tests for IfA virus were used systematically for hospitalised adult patients [26] and children [27], although these studies evaluated the impact of the result of the test and thus are not fully comparable.

The limitations of this study include the choice of antibiotic prescription as the primary outcome and its open-label design, which might have led to performance bias; that is, the physicians might have been influenced by the randomisation in making the decision whether to prescribe antibiotics. Also, on the basis of the study design with an optional follow-up visit, a large number of patients were lost to follow-up, and because of the relatively short follow-up period, the effect and duration of antibiotic treatment in relation to the diagnosis could not be properly evaluated. To influence antibiotic resistance and adverse events following antibiotic therapy, it is necessary to reduce the total rate of antibiotic prescriptions, which was not achieved in our study. However, at the time of planning for the study, limited prospective data were available on the performance of a multiplex PCR panel for the diagnosis of viral and bacterial ARTIs in clinical practise. It was not possible to define whether patients should be prescribed an antibiotic depending on the test result. We therefore chose antibiotic prescriptions at the initial visit as a straightforward primary outcome measure to evaluate whether access to the test would have any effect on antibiotic prescription rates, and this should be included in future studies of algorithms for the management of patients with ARTIs.

Systematic testing for bacterial pathogens such as *Streptococcus pneumoniae, Haemophilus influenzae *and *Moraxella catarrhalis *was not conducted in this study. These bacteria may act as potential respiratory pathogens (PRPs) harbouring in the upper respiratory tract, and an extension of the panel to include these bacterial agents might improve the clinical utility of the test. However, the interpretation of the detection of bacterial pathogens in nasopharyngeal samples in heterogeneous syndromes such as ARTIs is unclear. Cultured *S. pneumoniae *from the nasopharynx of adults has been shown to have high specificity but low sensitivity for the diagnosis of pneumococcal pneumonia [28]. PCR-based detection of *S. pneumoniae *from the oropharynx and nasopharynx without codetection of other commensal *Streptococcus *spp. poses technical difficulties.

Lieberman *et al*. [29] reported higher sensitivity in using nasopharyngeal washing compared to nasopharyngeal swabs in the detection of PRPs. To keep sampling simple, nasopharyngeal washing was not used in this study, which may constitute a study limitation. However, we used flocked nasopharyngeal swabs, which probably yield higher detection rates than previously reported in studies in which cotton-tipped nasopharyngeal swabs were used [30]. The study population consisted of patients with a broad spectrum of clinical manifestations, many of whom had mild symptoms. However, since the prescription of antibiotics for these patients is not uncommon, this was a deliberate study design.

Previously, Oosterheert *et al*. [18] investigated the impact of a PCR method for aetiologic diagnosis of lower RTIs in adults but failed to show any reduction in the use of antibiotics. However, their study included only hospitalised patients, and the end point was to register a change of ongoing treatment regimen rather than whether to make the initial decision to prescribe antibiotic treatment.

At follow-up, our study no longer showed any significant difference in antibiotic use between the two study groups. However, since a large number of patients were lost to follow-up, the selection of patients may have been biased. Moreover, physicians outside the study prescribed antibiotics for some patients between the initial visit and the follow-up visit. Because of the study design, the accuracy of prescriptions during follow-up could not be properly evaluated. Thus, our results must be interpreted with caution. In the absence of an algorithm to determine how to follow the patients and act upon the results of PCR testing, including a predefined antibiotic management plan, the observed reduction of antibiotic use at the initial visit may be lost by the time of follow-up. However, our study represents a proof of concept that access to a rapid etiologic diagnostic tool may affect therapeutic decision in this setting.

The antibiotic prescription rate in our study was low. A recent retrospective study of primary care patients in a comparable Swedish region recorded an antibiotic prescription rate of 45% for all patients with ARTIs, 60% for patients with acute bronchitis and 16% for patients with the 'common cold' [13]. The low prescription rate in our series could be due partly to a tendency to adhere more strictly than usual to current guidelines for the restrictive use of antibiotics.

We detected an infectious agent in 47% of patient samples taken at the initial visit, which is in line with previous studies in which detection rates between 43% and 63% in adults have been described [11,31]. Detection of a virus does not exclude concomitant bacterial infections or other noninfectious causes. This safety issue was discussed at the beginning of our study, and physicians were encouraged to treat bacterial complications at their own discretion. No SAEs were reported, but because of the relatively large number of patients lost to follow-up and the short duration of the study, we cannot exclude the possibility that some patients experienced late SAEs. Patient-relevant outcomes, such as the severity of symptoms over time, were not recorded, which is another limitation of this study. We deliberately chose not to include such outcomes, as the primary objective of the study was to evaluate the impact on antibiotic prescription rates at the initial visit. This study design also reduced the resources needed to conduct the study.

Analysis of CRP is frequently used to discriminate viral from bacterial infections [32]. Prescription rates increase with rising CRP levels [32,13]. In the group of patients with CRP levels > 100 mg/L, approximately one-third carried a respiratory virus, supporting the need for reliable tools for the aetiologic diagnosis of ARTIs. Distinguishing a viral from a bacterial aetiology in ARTIs is difficult on clinical grounds alone, and the predictive value of vital signs, CRP and X-ray findings is low [7,8]. Procalcitonin (PCT) has been proven to be useful in reducing antibiotic prescriptions in hospitalised patients with lower RTIs [33,34], whereas in primary care, the evidence of PCT for this purpose is more ambiguous. One study of adults with ARTI (one to twenty-eight days' illness duration) judged to be in need of antibiotics and treated as outpatients reported a positive effect of antibiotic use [35], but another study of children with community-acquired pneumonia, a significant proportion of whom were treated in the hospital, showed a negative effect [36].

No distinct pattern of symptoms that could guide the clinician towards a correct aetiologic diagnosis was identified in our study. Vomiting, which was the only independent variable predicting antibiotic use, may be interpreted as a sign of serious illness justifying antibiotic treatment.

Reducing the unnecessary use of antibiotics in the treatment of patients with ARTIs is of utmost importance and cannot be accomplished by using a single strategy. Patient- and physician-oriented educational programmes could play an important role as previously suggested [37]. However, these programmes do not solve the issue of the lack of an aetiologic diagnosis, which is important not only for adequate use of antibiotics but also for addressing issues such as possible complications, prognosis, antiviral treatment options, surveillance and infection control.

## Conclusions

In conclusion, we have shown that access to a multiplex RT-PCR assay for the aetiologic diagnosis of ARTIs may reduce the prescription rate of antibiotics at the initial visit in an outpatient setting. To sustain the effect, it seems necessary to define how to follow and manage the patient according to the result of the test, which warrants further investigation. We believe that the implementation of similar methods in routine clinical care may be a useful tool to reduce the overprescription of antibiotics in patients with ARTIs.

## Competing interests

The authors declare that they have no competing interests.

## Authors' contributions

RBL contributed to the study design, data collection, data analysis and the writing of the manuscript and was the main person responsible for database management. JW and LMA contributed to the study design, data collection, data analysis and the writing of the manuscript. SO and ML contributed to the study design, technical issues and the writing of the manuscript. All authors read and approved the final manuscript.

## Pre-publication history

The pre-publication history for this paper can be accessed here:

http://www.biomedcentral.com/1741-7015/9/44/prepub
